# HIV Infection and Cardiovascular Disease

**DOI:** 10.1155/2013/252463

**Published:** 2013-12-30

**Authors:** Sandra C. Fuchs, Marina Beltrami-Moreira, Bolanle Oyeledun, Papa Salif Sow, Marco Vitoria

**Affiliations:** ^1^Postgraduate Studies Program in Cardiology, School of Medicine, Universidade Federal do Rio Grande do Sul, R. Ramiro Barcelos 2600, 90035-003 Porto Alegre, RS, Brazil; ^2^National Institute for Health Technology Assessment (IATS/CNPq), Hospital de Clínicas de Porto Alegre, R. Ramiro Barcelos 2350, 90035-003 Porto Alegre, RS, Brazil; ^3^Department of Nutrition, Harvard School of Public Health, 677 Huntington Avenue, Boston, MA 02115, USA; ^4^Harvard Medical School, New Research Building, Avenue Louis Pasteur, Room 724, Boston, MA 02115, USA; ^5^Centre for Integrated Health Programs (CIHP), Plot 1129 Kikuyu Close, Off Nariobi Street, Wuse II Abuja, FCT, Nigeria; ^6^ICAP, Mailman School of Public Health Columbia University, 722 West 168th Street, New York, NY 10032, USA; ^7^University of Dakar, Department of Infectious Diseases Fann Hospital, BP 5035, Dakar, Senegal; ^8^HIV/AIDS Department, World Health Organization, 20, Avenue Appia, CH-1211 Geneva 27, Switzerland

HIV infection has been considered as one of the major global public health threats of the last century, increasing several times the mortality rate in comparison to the cholera epidemic that swept London in the nineteenth century. Unlike the contamination caused by cholera bacillus, prevention of HIV infection requires other interventions than basic sanitation, and the antiretroviral agents currently remain the touchstone to transform the disease from subacute into a chronic condition. The introduction of highly active antiretroviral therapy (HAART) in the treatment of HIV-infected patients, in 1990s, has allowed better health status and greater longevity among people living with HIV, associated with a reduction in the viral transmission rate. For a while, it has been believed that universal access to HAART and the progressive increase in the efficiency of the prevention methods could save future generations [1]. However, it requires an enormous effort to expand HIV testing, maintenance of sustained treatment, and implementation of prevention programs. Nevertheless, HAART is not free of adverse effects.

With the significant reduction of the mortality and morbidity associated with HIV induced immunodeficiency in patients using HAART, the HIV infection is behaving as a long-term sickness [2]. HIV infection and its treatment have been associated with abnormal metabolic profile [3]; increased prevalence of noncommunicable diseases [4, 5] and mortality rate of AIDS-related have shifted to non-AIDS-related conditions [6, 7]. In the pre-HAART era, mortality from cardiovascular disease in infected persons occurred almost two decades earlier than in the general population. After the introduction of HAART, this difference went on to about nine years [8]. Even though the HIV-infected population is getting older, most still ar less than 50 years old and, in the United States, only half of the population will be 50 years in 2015 [9]. Therefore, the long-term management of patients with HIV infection has to be expanded to diagnosis, treatment, and prevention of cardiovascular risk factors and coronary heart disease. Even so, most current guidelines for the treatment of HIV infection are still focused only on antiretroviral treatment and do not take into account the treatment and prevention of comorbidities not related to AIDS [10–12]. However, bringing the paradigm of cardiovascular disease prevention for the scenario of HIV infection requires detection of the prevalence of cardiovascular risk factors and coronary heart disease in HIV-infected patients.

In this edition of this journal, a portrait of the Brazilian scenario of cardiovascular disease among HIV-infected patients was presented. Brazil is a country that has provided free access to HIV treatment for the entire population of infected people in the last two decades, and the use of HAART had a great impact on the costs of health care and the demand for the public health system. D. V. Araújo et al. showed that, in the last five years, the number of HIV/AIDS cases increased approximately by 40%, among patients under 50-years of age, yet the hospital admissions due to AIDS remained stable. Conversely, there was a marked increase in the hospitalizations due to acute myocardial infarction. R. K. Lazzaretti et al. provided data on genetic basis for understanding the complexity of the dyslipidemia in HIV infection. They detected that single nucleotide polymorphisms in six candidate genes (APO B, APO A5, APO E, APO C3, SCAP, and LDLR) were associated with dyslipidemia, showing that genetic factors contribute to determining the lipid profile in HIV-infected individuals on antiretroviral therapy.

Even so, there is a lack of robust evidence for prescribing agents to reduce dyslipidemia in HIV-infected patients. The new guidelines for cholesterol treatment highlighted the lack of randomized clinical trials on the potential benefits of statin therapy to reduce the risk of atherosclerotic cardiovascular disease in HIV-infected patients exceeding the risk of adverse events or drug interactions [13].

Another approach, previously described for the general population [14, 15], was to determine whether the association between consumption of alcoholic beverages and hypertension was modified by race in HIV-infected individuals. Among lifestyle characteristics, the consumption of large amounts of alcohol was independently associated with hypertension in white and nonwhite HIV-infected individuals. M. L. R. Ikeda et al. showed that there was an association of blood pressure with the frequency of consumption among the whites, while for nonwhite participants the amount of alcohol consumed was more important than the pattern of consumption in raising blood pressure. Although some of lifestyle characteristics are not modifiable, alcohol consumption is suitable for intervention.

In an attempt to compare some tools available to assess the overall cardiovascular risk profile of HIV-infected patients, M. W. Nery et al. calculated the traditional Framingham risk score, the Prospective Cardiovascular Münster (PROCAM) score; both originally developed for non-HIV-infected population; and the Data Collection on Adverse Effects on Anti-HIV Drugs (DAD) score, validated on HIV-infected patients. They found that the proportion of patients classified as being at moderate risk or higher was larger for the Framingham than for the PROCAM score. While these results have clinical follow-up and management implications, there was no comparison with data collected for the incidence of events. The use of Framingham score seems to have the advantage of allowing the comparison with other studies conducted in non-HIV-infected population [16]. Finally, a pooled analysis carried out in three cities of the Northeast, Midwest, and Southern Brazil showed that, irrespective of HIV status or treatment, classically risk-associated conditions, such as hypertension and diabetes, persist as the most relevant risk factors for cardiovascular disease. Moreover, these conditions were present at a younger age in the studied population. Of note is also the high prevalence of moderate and high risk according to the Framingham risk score among women. In this group, the diagnosis of cardiovascular disease and ischemic heart disease frequently occurs later than in men. This study reminds us that it is never too early to approach these problems and emphasize primary prevention of cardiovascular disease, even among populations with chronic conditions such as the HIV infection. We believe that the spectrum of cardiovascular manifestations among patients infected by HIV, pictured in this edition, allows the design and implementation of initiatives aimed at controlling and preventing the impact of cardiovascular disease.

*Sandra C. Fuchs* *Sandra C. Fuchs* *Marina Beltrami-Moreira* *Marina Beltrami-Moreira* *Bolanle Oyeledun* *Bolanle Oyeledun* *Papa Salif Sow* *Papa Salif Sow* *Marco Vitoria* *Marco Vitoria*
